# Sweetening Pharmaceutical Radiochemistry by ^18^F-Fluoroglycosylation: Recent Progress and Future Prospects

**DOI:** 10.3390/ph14111175

**Published:** 2021-11-17

**Authors:** Sandip S. Shinde, Simone Maschauer, Olaf Prante

**Affiliations:** Department of Nuclear Medicine, Molecular Imaging and Radiochemistry, Friedrich-Alexander University Erlangen-Nürnberg (FAU), D-91054 Erlangen, Germany; shinde88@gmail.com (S.S.S.); simone.maschauer@uk-erlangen.de (S.M.)

**Keywords:** fluorine-18, prosthetic group, ^18^F-fluoroglycosylation, positron emission tomography, PET

## Abstract

In the field of ^18^F-chemistry for the development of radiopharmaceuticals for positron emission tomography (PET), various labeling strategies by the use of prosthetic groups have been implemented, including chemoselective ^18^F-labeling of biomolecules. Among those, chemoselective ^18^F-fluoroglycosylation methods focus on the sweetening of pharmaceutical radiochemistry by offering a highly valuable tool for the synthesis of ^18^F-glycoconjugates with suitable in vivo properties for PET imaging studies. A previous review covered the various ^18^F-fluoroglycosylation methods that were developed and applied as of 2014 (Maschauer and Prante, BioMed. Res. Int. 2014, 214748). This paper is an updated review, providing the recent progress in ^18^F-fluoroglycosylation reactions and the preclinical application of ^18^F-glycoconjugates, including small molecules, peptides, and high-molecular-weight proteins.

## 1. Introduction

Positron emission tomography (PET) is a highly sensitive medical imaging technique that relies on the use of radioactive tracers for the quantification of biochemical processes in vivo. While various radionuclide positron emitters are suitable for PET, fluorine-18 gained highest interest as the PET radionuclide of choice due to its superior characteristic features of energy (E_max_(β^+^) = 635 keV) and half-life (t_1/2_ = 109.7 min), which allow for multistep radiochemical syntheses and transportation from production centers to external radiopharmacies [1]. In general, fluorine is one of the highly demanding halogen atoms in medicinal and pharmaceutical chemistry due to its unique physicochemical properties [2]. The exchange of a hydrogen atom by fluorine in a biomolecule can improve the biochemical properties of the molecule significantly, which has, in turn, an effect on the membrane permeability, metabolic stability, (improved) solubility, and receptor-interaction properties [3]. Numerous methods and reaction conditions were developed to facilitate the synthesis of fluorinated molecules by nucleophilic aliphatic and aromatic substitution [4,5], including radiochemical approaches but also non-radioactive chemistry suggesting their application in radiopharmaceutical chemistry [6,7]. Moreover, the introduction of ^18^F in bioactive molecules by the use of ^18^F-labeled prosthetic groups is frequently achieved by applying chemoselective strategies for a straightforward design of new PET tracers [8].

Based on Hamacher’s synthesis of β-d-mannopyranose triflate [9] as a precursor for the highly efficient radiosynthesis of 2-[^18^F]-fluoro-2-deoxy-D-glucose ([^18^F]FDG, [10]), being the major driving force in the emerging field of PET in nuclear medicine, the idea of using [^18^F]FDG or derivatives of [^18^F]FDG for chemoselective ^18^F-fluoroglycosylation reactions has been followed over the years. The ^18^F-fluorglycosylation approach aims at a chemoselective and mild labeling method, simultaneously providing the opportunity to influence the biodistribution and tracer uptake characteristics by the introduction of the hydrophilic glycosyl group. It is well known that the glycosylation of biomolecules, such as peptides or proteins, could improve their in vivo stability in blood and accelerate the clearance of distinct glycoconjugates through the kidneys [11,12,13]. Additionally, a series of previous publications have shown that glycosylation prior to radiolabeling was beneficial for improved in vivo properties of several peptide-based PET tracers [12,13,14,15,16]. As one of the most commonly known chemoselective synthetic strategy, the “click chemistry” concept by Sharpless and co-workers [17] was also widely applied in carbohydrate chemistry, facilitating the synthesis of a wide variety of glycoconjugates [18]. Therefore, previous work in our research group was concerned with a click chemistry-based ^18^F-fluoroglycosylation strategy, starting from a series of mannosyl azide precursors [19] and implementing a convenient approach to the radiosynthesis of ^18^F-labeled glycopeptides as effective imaging agents for PET [20]. Since then, we and others have frequently applied different ^18^F-fluoroglycosylation approaches to the radiosynthesis of various ^18^F-labeled glycoconjugates as PET tracers. A first review article on ^18^F-fluoroglycosylation reactions was published in 2014 [21]. In the present review, we provide an update on the various ^18^F-fluoroglycosylation methods and strategies which were developed and adapted to the synthesis of various ^18^F-glycoconjugate tracers for PET over the past decade.

Table 1 provides an overview of a selection of ^18^F-labeled glycosyl derivatives that are used as prosthetic groups for the radiosynthesis of ^18^F-glycoconjugates as potential PET tracers. The following subchapters provide some examples for their application with a focus on recent work published since 2014. In this review, the terms radiochemical yield (RCY) and radioactivity yield (RAY) were used by following the “Consensus nomenclature rules for radiopharmaceutical chemistry” [22]. The RCY is corrected for radioactive decay and usually determined by radio-high performance liquid chromatography (HPLC) analysis of the reaction solution, while the RAY is not decay-corrected and defined as the percentage of radioactivity of the product relative to the starting radioactivity of [^18^F]fluoride.

## 2. 2-Deoxy-2-[^18^F]fluoro-β-glucosyl Azide for Click Chemistry Based ^18^F-Fluoroglycosylation

The Cu(I)-catalyzed Huisgen 1,3-cycloaddition reaction of an azide and an acyclic alkyne (CuAAC) to yield a 1,2,3-triazole is one of the most prominent reactions belonging to the concept of “click chemistry” [17], defining reactions that are easy to perform, high-yielding, chemoselective, orthogonal and proceed without the formation of by-products. The successful adaption of CuAAC to ^18^F-chemistry taking advantage of high selectivity, reliability, fast and mild reaction conditions had already been amply documented [29].

**Scheme 1:**
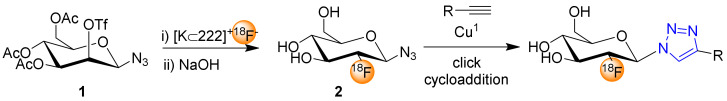
Two-step ^18^F-fluoroglycosylation by click cycloaddition using 2-deoxy-2-[^18^F]fluoroglucopyranosyl azide (**2**), starting from the 2-*O*-triflate precursor of triacetylated β-mannosyl azide (**1**).

Scheme 1 shows the synthesis of the ^18^F-fluoroglycosylating agent 3,4,6-tri-*O*-acetyl-2-deoxy-2-[^18^F]fluoroglucopyranosyl azide (**2**), that was achieved by the ^18^F-labeling of mannosyl precursor 3,4,6-tri-*O*-acetyl-2-*O*-trifluoromethanesulfonyl-β-d-mannopyranosyl azide (**1**, Scheme 1) in a high radiochemical yield (RCY) of 71%, as demonstrated by Maschauer and Prante in 2009 [19]. Interestingly, the RCY of the ^18^F-substitution depended mainly on the chemical purity of the mannosyl precursor after recrystallization in ethanol, an observation that is similar to the well-known [^18^F]FDG synthesis. Radiolabeling of β-mannosyl azide **1** was performed under standard conditions (Kryptofix 222, K_2_CO_3_) or with K_2_CO_3_/KH_2_PO_4_ under less basic conditions to reduce the degradation of β-mannosyl azide and thereby simplifying the HPLC purification of **1**.

**Figure 1:**
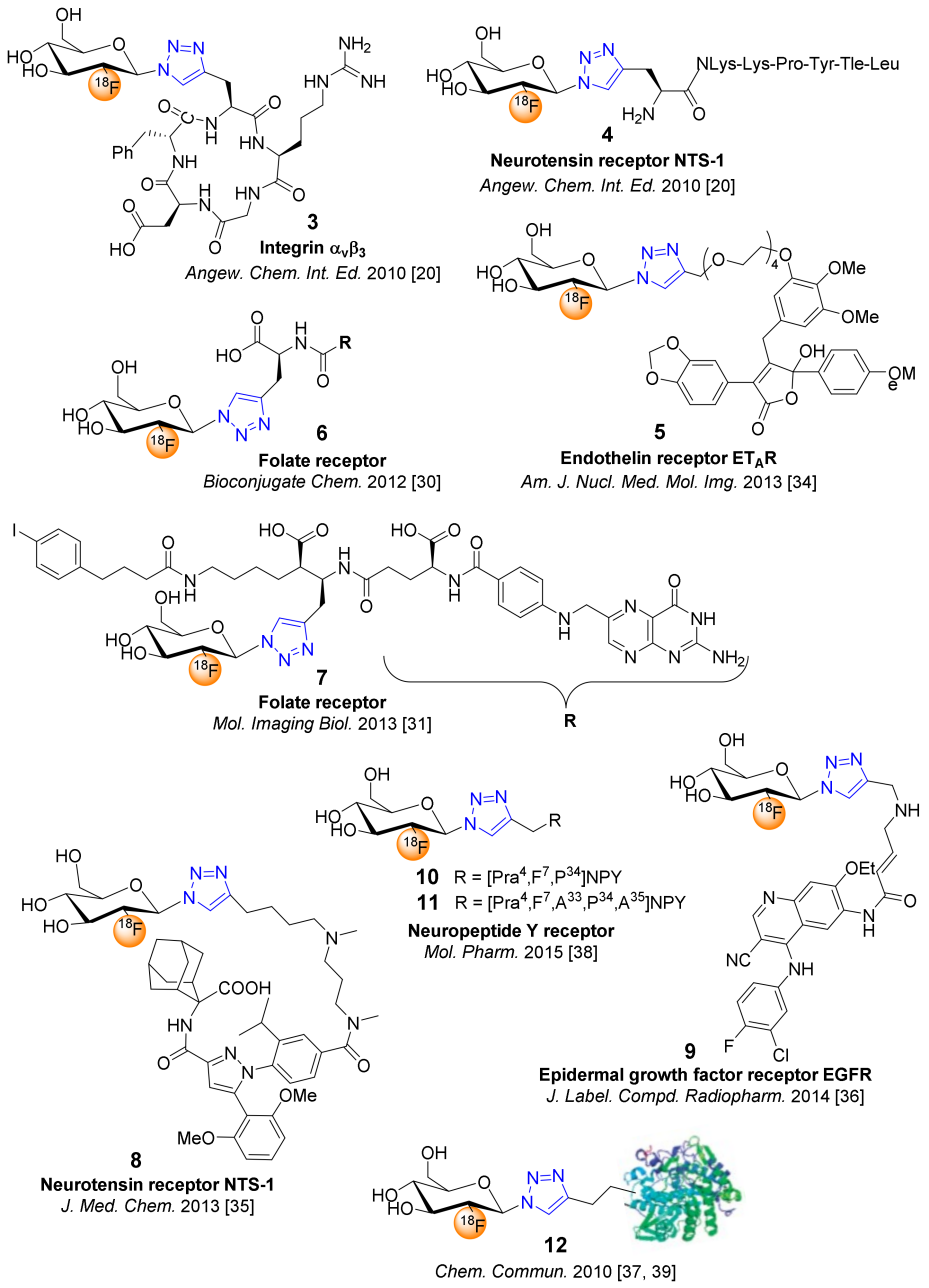
Click chemistry-based 2-deoxy-2-[^18^F]fluoroglycosylated ligands and their target receptors.

The application of the prosthetic group **2** for CuAAC was initially successfully optimized for alkyne-bearing amino acids [19] and then applied to the radiosynthesis of ^18^F-glycopeptides, namely an RGD glycopeptide for PET imaging of integrin α_v_β_3_ (**3**) and a neurotensin peptoid for PET imaging of neurotensin receptor 1 (NTS1)-positive tumors (**4**) (Figure 1) [20]. The optimized click reaction was performed in PBS/EtOH (10:1) at 60 °C containing 0.2 mM peptide alkyne in the presence of CuSO_4_ (4 mM) and sodium ascorbate (12 mM). The ^18^F-glycopeptides were isolated by HPLC in a 17–20% radioactivity yield (RAY) after a total synthesis time of 70–75 min with 55–210 GBq/µmol molar activities and subjected to tumor-bearing nude mice for the successful characterization of in vivo specificity by small animal PET [20].

Moreover, the prosthetic glycosyl azide **2** was also applied to ^18^F-fluoroglycosylation of various non-peptidic molecules [30,31,32,33,34,35,36]. Interestingly, Fischer et al. reported the radiosynthesis of an ^18^F-fluoroglycosylated folate, using solid phase extraction of the intermediate 3,4,6-tri-*O*-acetyl-2-deoxy-2-[^18^F]fluoroglucopyranosyl azide, thereby omitting the more laborious HPLC purification after the ^18^F-substitution reaction [30]. The CuAAC with folate alkyne proceeded in aqueous EtOH (38%) in the presence of Cu(OAc)_2_ (1.2 mM) and sodium ascorbate (2.4 mM) and ^18^F-glycofolate **6** was achieved after final HPLC purification in RAY of up to 25%, with a molar activity of 90 ± 38 GBq/μmol. Analyses of tissue samples at 30 min post-injection (p.i.) in mice confirmed a high stability of **6** in vivo and small-animal PET studies demonstrated that **6** showed high specific uptake and retention in folate receptor-positive tumors, together with fast blood clearance (tumor-to-blood ratio: 36 ± 15 at 90 min p.i.). The introduction of an albumin binding moiety to the folate precursor, in order to enhance of the blood circulation time of the glycoconjugate tracer, and CuAAC with **2** in the presence of Cu(OAc)_2_ (1 mM) and sodium ascorbate (3 mM) in water / DMF (60:40) at 50 °C for 15 min gave **7** in a RCY of 15% [31]. The RAY of **7** was only 1–2 % after a total synthesis time of 3 h in molar activities of 20 to 50 GBq/μmol. As expected, **7** revealed a slow blood clearance with tumor uptake values of 11–15 %ID/g at 1–4 h p.i. in PET studies of KB tumor-bearing nude mice and a substantially improved tumor-to-kidney ratio of about 1.

The ^18^F-fluoroglycosylation by CuAAC applying **2** for small molecules was also used for the radiosynthesis of a subtype-selective glycosylated ligand **5** for the endothelin receptor (ET_A_R) [34], the non-peptidic neurotensin receptor (NTS1) ligand **8** [35], and the fluoroglycosylated cyanoquinoline **9** as a PET ligand candidate for the epidermal growth factor receptor (EGFR) [36] (Figure 1).

The CuAAC for glycoconjugate **5** (alkyne (0.6 mM), sodium ascorbate (12 mM), CuSO_4_ (4 mM) in saline/EtOH (3:2)) gave high RAY (20–25%, 70 min) and **5** demonstrated high metabolic stability in vivo, fast blood clearance, low uptake in the kidneys and liver, but a very high uptake in the bile and intestines [34]. Therefore, glycoconjugate **5** is an example of a glycoconjugate that is predominantly excreted via hepatobiliary clearance, such that glycosylation did not significantly change the excretion pathway of analogs of the lead compound PD 156707.

Similarly, the ^18^F-fluoroglycosylation of a diarylpyrazole, derived from the potent NTS1 antagonist SR142948A, was also successfully performed by CuAAC of **2** with the alkyne-bearing diarylpyrazole precursor (0.3 mM) in saline/THF (3:4) for 10 min at 60 °C [35]. The ^18^F-glycoconjugate **8** was obtained in a RAY of 20 ± 3% and a molar activity of 35−74 GBq/μmol in a total synthesis time of 70 min. Glycoconjugate **8** displayed excellent NTS1 affinity (K_i_ = 1 nM) in vitro, high stability in vivo, rapid clearance from blood in vivo, and PET studies in nude mice bearing HT29 tumors demonstrated specific tracer uptake and excellent tumor retention with a tumor-to-blood ratio of 4.4 at 60 min p.i.

The ^18^F-fluoroglycosylation applying CuAAC with glycosyl azide **2** as a prosthetic group was also adopted to the radiosynthesis of a 4 kDa neuropeptide Y analog (**10**) and a high-molecular-weight ^18^F-glycoprotein (**12**) [37,38]. However, the Davies group employed the click conjugation of **2** with a very low concentration of the alkyne-bearing protein (6 µM) in the presence of Cu(I)Br and TTMA (triethyl 2,2′,2′’-[nitrilotris(methylene-1*H*-1,2,3-triazole-4,1-diyl)]triacetate) at room temperature, where the RCY of the ^18^F-glycosylated protein (**12**) was limited to 4.1% [37]. Thus, ^18^F-fluoroglycosylation of proteins by CuAAC using **2** is not well suited for proteins that are not readily available. The thionation of [^18^F]FDG offers [^18^F]FDG-SH as an alternative prosthetic group for ^18^F-fluoroglycosylation of proteins [39]; however, ^18^F-labeled glycoproteins remain particularly rare.

Based on many efforts in the design of neuropeptide Y (NPY) peptide analogs for studying the neuropeptide Y Y_1_ receptor (Y_1_R) in breast cancer, Hofmann et al. reported a ^18^F-fluoroglycosylated peptide for imaging Y_1_R-positive tumors by small-animal PET [38]. Applying **2** for the click chemistry based strategy of the fluoroglycosylated (FGlc) peptide analogue [Pra^4^(FGlc),F^7^,P^34^]NPY, the alkyne-bearing propargylglycine (Pra) peptide [Pra^4^,F^7^,P^34^]NPY was synthesized and subjected to ^18^F-fluoroglycosylation, affording a RAY of 20−25% and molar activity of 40−70 GBq/μmol in a total synthesis process of 75 min. The glycosylated peptide [Pra^4^(FGlc),F^7^,P^34^]NPY (**10**) demonstrated subtype selectivity for Y_1_R over Y_2_R and a high potency for the induction of Y_1_R-mediated inositol accumulation in vitro (EC_50_ = 3.1 nM). In vitro autoradiography with Y_1_R-positive MCF-7 tumor tissue slices indicated a high specific binding of the ^18^F-labeled glycopeptide, when binding was reduced by 95% ([Pra^4^,F^7^,P^34^]NPY) and by 86% (BIBP3226, Y_1_R antagonist) in competitive binding studies. Small-animal PET studies with [Pra^4^([^18^F]FGlc),F^7^,P^34^]NPY (**10**) on MCF-7 breast tumor-bearing nude mice in direct comparison with a scrambled low-affinity peptide (**11**, Figure 1) revealed specific uptake of **10** in the MCF-7 tumor with increasing tumor-to-blood ratio from 1.2 to 2.4, a tumor retention of 76 % (45−90 min p.i.) and decreased kidney uptake compared to DOTA analogues of this peptide. The ^18^F-glycopeptide [Pra^4^([^18^F]FGlc),F^7^,P^34^]NPY (**10**) can be considered as a lead peptide for the design of improved glycopeptide tracers with shorter amino acid sequences for imaging of Y_1_R-positve breast tumors by PET [38].

## 3. 6-Deoxy-6-[^18^F]fluoro-β-glycosyl Azides for Click Chemistry-Based ^18^F-Fluoroglycosylation

**Scheme 2:**
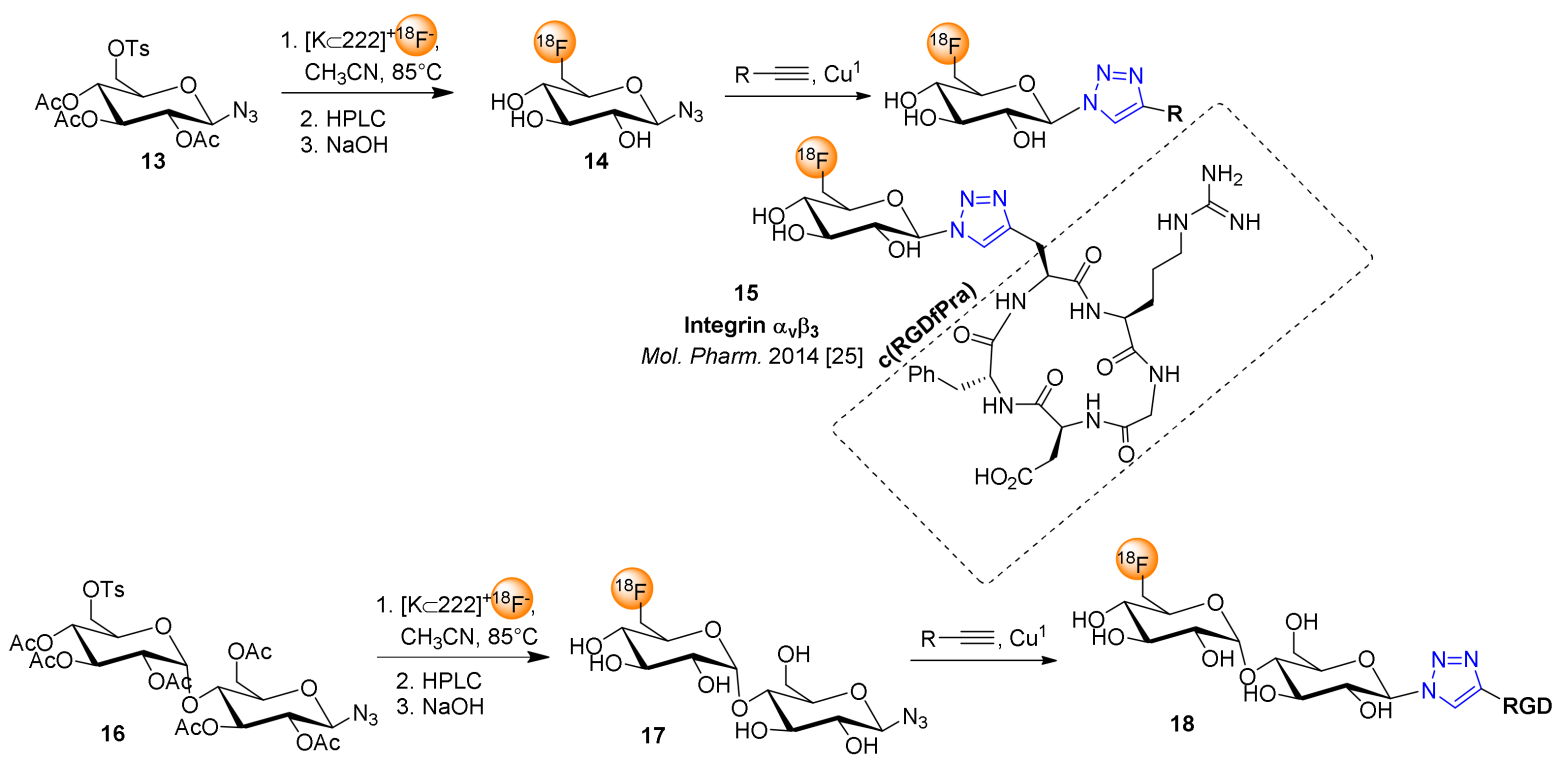
^18^F-fluoroglycosylation by CuAAC using 6-deoxy-6-[^18^F]fluoroglucopyranosyl azide **14** or 6´-deoxy-6´-[^18^F]fluoromaltosyl azide **17** with the cyclic peptide c(RGDfPra) alkyne (according to [26]).

Scheme 2 depicts the application of the prosthetic groups 6-deoxy-6-[^18^F]fluoroglucopyranosyl azide (**14**) and 6′-deoxy-6’-[^18^F]fluoromaltosyl azide (**17**) for click ^18^F-fluoroglycosylation of an RGD peptide [26]. Both ^18^F-glycosides were synthesized from their peracetylated 6-tosylate precursors **13** and **16** in high RCY of 84% and 61%, respectively. The resultant intermediates were purified by HPLC and subsequently hydrolyzed with NaOH (60 mM) to give the glycosyl azides **14** and **17**, that were conjugated to the cyclic peptide c(RGDfPra) by CuAAC under similar reaction conditions as described above for **1**. Following this strategy, 6-[^18^F]FGlc-RGD (**15)** and 6′-[^18^F]Mlt-RGD (**18**) were achieved in RAY of 16−24% and molar activities of 50–200 GBq/µmol within 70–75 min. A comparative PET study demonstrated that both ^18^F-glycopeptides **15** and **18** showed significantly decreased liver and kidney uptake relative to 2-[^18^F]FGlc-RGD (**3**) in vivo using U87MG tumor-bearing nude mice [26]. Importantly, the maltosyl peptide **18** revealed substantial tumor uptake and high tumor retention comparable to that of ^18^F-galacto-RGD [14,40] and high tumor-to-kidney ratios comparable with dimeric RGD peptides [41,42], such that the high tumor uptake and excellent clearance properties in vivo make **18** an alternative glycopeptide tracer for imaging integrin expression by PET.

**Figure 2:**
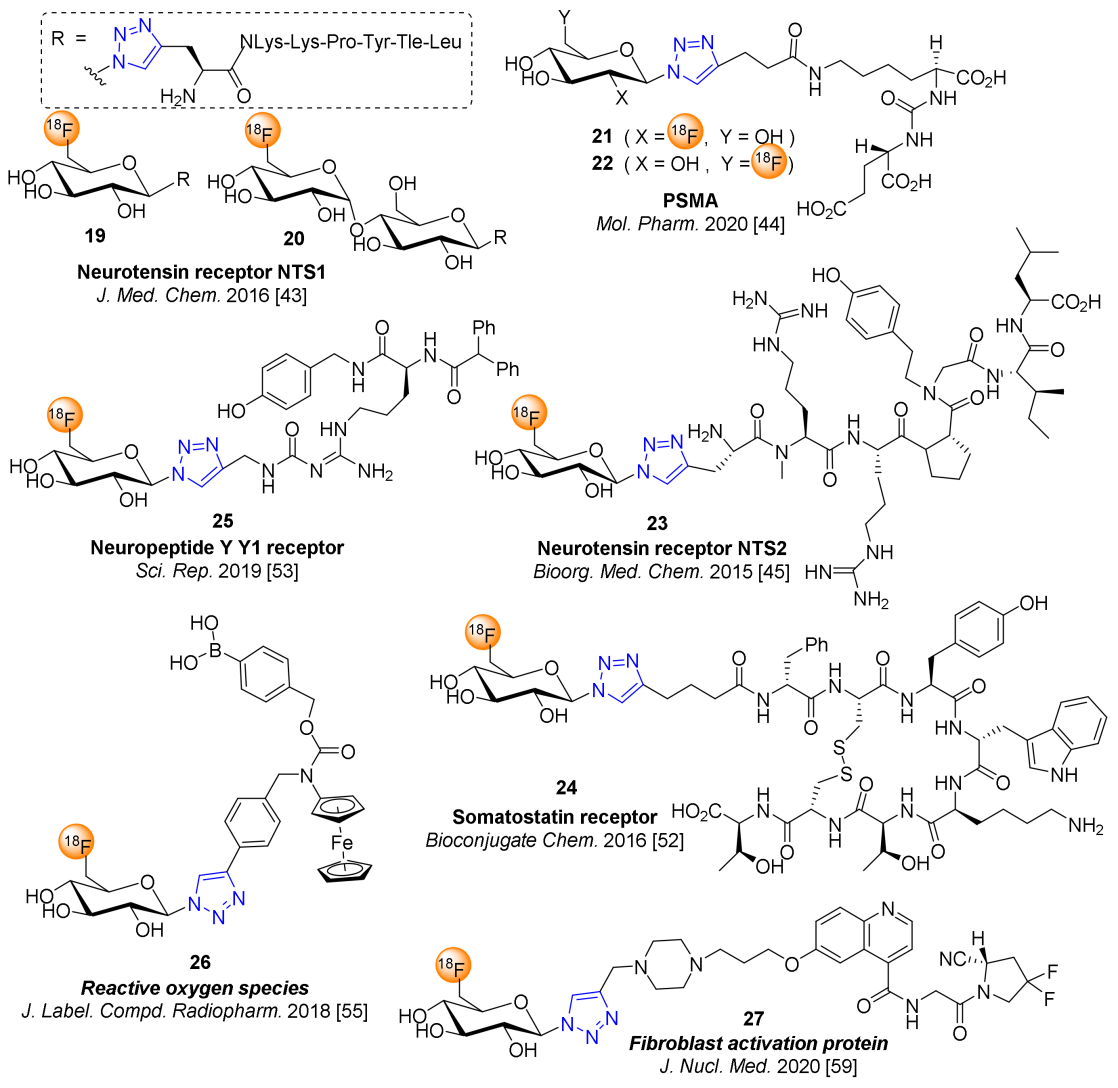
Overview of PET tracers synthesized via CuAAC using the ^18^F-labeled prosthetic group 6-deoxy-6-[^18^F]fluoro-β-glucosyl azide (**14**). For a direct comparison (see text), the structures of 6′-deoxy-6′-[^18^F]fluoromaltosyl peptide **20** and 2-deoxy-2-[^18^F]fluoroglucosyl peptide **21** are included.

Inspired by the promising results of 6-[^18^F]FGlc-RGD (**15**), especially in terms of clearance through the kidneys, we extended the application of 6-deoxy-6-[^18^F]fluoroglucopyranosyl azide (**14**) for ^18^F-fluoroglycosylation of a series of bioactive compounds shown in Figure 2.

Thus, with the aim of improving the renal clearance of **4** (see Figure 1), the influence of fluoroglycosylation of the NTS1-affine linear peptoid PraNLysLysProTyrTleLeu was investigated [43]. The NTS1 affinity of the target compounds **19** and **20** (Figure 2) were 26 nM and 33 nM, respectively, which compares very well with the best ^68^Ga-labeled analogues. The ^18^F-fluoroglycosylation of the N-terminally propargylglycine (Pra)-derivatized peptoids according to Scheme 2 occurred in good RAY of 16–21% after a total synthesis time of 80–85 min. The biodistribution studies of **19** and **20** in HT29 tumor-bearing mice showed significantly better renal clearance compared to **4** and ^68^Ga-labeled peptoids, but a 40% reduced uptake in the tumor [43].

The click chemistry strategy for ^18^F-fluoroglcosylation using 2-deoxy-2-[^18^F]fluoroglucopyranosyl azide (**2**) or 6-deoxy-6-[^18^F]fluoroglucopyranosyl azide (**14**) was applied to prostate-specific membrane antigen (PSMA) inhibitors of the glutamate-urea-lysine type to afforded 2-[^18^F]FGlc-PSMA (**21**) and 6-[^18^F]FGlc-PSMA (**22**) [44]. The ^18^F-fluoroglycosylated PSMA inhibitors **21** and **22** were afforded in RAY of 19−22% and with molar activities of 71−136 GBq/μmol. The PSMA inhibitory potencies were moderate for **21** (IC_50_ = 234 nM) and **22** (IC_50_ = 59 nM). Small animal PET studies using PSMA-positive PC-3 PIP and PSMA-negative PC-3 tumor-bearing nude mice revealed specific uptake of **21** (13%ID/g) and **22** (6%ID/g) in PC-3 PIP tumors at 60 min p.i. Highly remarkably, **21** had high uptake in the kidneys with very high retention (74 to 72%ID/g at 30 to 60 min p.i.), while **22** showed very low uptake in the kidneys of 7.5%ID/g at 30 min p.i. with rapid clearance (0.9%ID/g at 120 min p.i.). Thus, the 6-fluoroglucosyl analog **22**, with an adequate uptake in PSMA-positive tumors, a considerably low kidney uptake and fast clearance from the kidneys, it could be a promising radiotracer for translation into the clinic [44].

The ^18^F-fluoroglycosylation via the clickable prosthetic group **14** was also applied to the first radiosynthesis of a neurotensin receptor 2 (NTS2)-subtype selective peptide ligand reported by Maschauer et al. [45] NTS2-selective PET ligands had not been previously described, such that the availability of a subtype selective NTS2 radioligand for PET could be a valuable tool for studying the role of this subtype in various tumor types including prostate, pancreas and breast carcinoma [46,47,48,49,50]. Maschauer et al. reported the radiosynthesis of an ^18^F-glycopeptoid accomplished by a modified CuAAC between the prosthetic group **14** and the alkyne-terminated NT(8–13) analog Pra-*N*-Me-Arg-Arg-Pro-*N*-homo-Tyr-Ile-Leu-OH. Very interestingly, the glycopeptide Pra(6FGlc)-*N*-Me-Arg-Arg-Pro-*N*-homo-Tyr-Ile-Leu-OH (**23**) revealed equal NTS2 affinity of K_i_ = 7 nM relative to the non-glycosylated sequence (*N*-Me-Arg-Arg-Pro-*N*-homo-Tyr-Ile-Leu-OH) [45,51]. Remarkably, the use of tris-(3-hydroxypropyltriazolylmethyl)amine (THPTA) in the CuAAC reaction with **14** significantly accelerated the formation of **23** and reduced the necessary amount of alkyne peptide precursor to 20 nmol. In vitro studies on rat brain slices revealed the subtype selectivity of ^18^F-glycopeptoid **23** for NTS2. As **23** displayed high stability in vitro but fast degradation in vivo, PET imaging experiments using HT29 and PC3 tumor-bearing nude mice revealed only moderate specific uptake of **23** in NTS2-positive tumors [45]. Further studies are needed for the development of more metabolically stable NTS2-selective peptides for PET.

Bioactive peptides are clearly a very important and prominent class of compounds that are highly suitable for the method of ^18^F-fluoroglcosylation. The synthetic octapeptide analogs derived from the native somatostatin peptides SST-14 and SST-28, namely octreotate (TATE) or octreotide (TOC), are high affinity ligands for the somatostatin receptors (sstr), preferably subtypes 2 and 5, which are overexpressed on neuroendocrine tumors (NET). The ^18^F-glyco-octreotate analog [^18^F]FGlc-TATE (**24**) was achieved by the “click”-^18^F-fluoroglycosylation using **14** in a RAY 19–22% and molar activities of 32−106 GBq/μmol [52]. The ^18^F-glycopeptide **24** showed high affinity to somatostatin receptors expressed on AR42J cells with fast and high internalization, and a beneficial logD_7.4_ of −1.8. In AR42J tumor bearing nude mice, small animal PET studies revealed high uptake of **24** in the tumor and fast clearance of **24** from other organs resulting in an excellent tumor-to-blood ratios of 17 at 60 min p.i. Therefore, ^18^F-glyco-octreotide **24** could be considered as a reliable alternative ^18^F-labeled radiopeptide for imaging somatostatin receptor-positive tumors by PET due to excellent in vitro and in vivo properties.

Similarly, ^18^F-glycoazide **14** was linked to an alkyne derivative of BIBP3226 to afford the fluoroglycosylated derivative **25** as a Y_1_R radioligand candidate for PET of breast cancer [53]. This study showed that the glycosyl derivative **25** displayed a highly decreased Y_1_R affinity of 208 nM when compared to the corresponding fluoroethoxyethyl derivative (2.8 nM). Consequently, despite its favorable hydrophilicity, **25** demonstrated low binding to human breast cancer MCF-7-Y1 cells and slices of tumor xenografts in vitro and was not suitable for the in vivo detection of Y_1_R-positive tumors by PET studies. The comparative study demonstrated that the corresponding ^18^F-fluoroethoxyethyl and ^18^F-PEGylated derivatives, despite their higher lipophilicity, were more promising than **25** and showed displaceable and specific binding to Y_1_R in vitro and in vivo [53].

Tracers for imaging the content of reactive oxygen species (ROS) in tumors could be valuable for PET imaging of tumors and contribute to our knowledge of the biodistribution of anticancer drug candidates that are ROS-dependently trapped in tumor cells [54]. A click chemistry-based ^18^F-fluoroglycoconjugation of N-alkylaminoferrocene as a potential anticancer agent was optimized by Toms et al., employing **14** and Cu(OAc)_2,_ phosphate buffer/THF, and sodium ascorbate for the CuAAC reaction conditions [55]. Noteworthily, the purification of the ^18^F-labeled aminoferrocene glycoconjugate was problematic, since the hydrolysis of the boronic acid ester and oxidation of non-carrier-added **26** occurred in the buffered solution. However, the RCY (referred to the CuAAC reaction) of carrier-added **26** was 85% under optimized conditions [55]. Further PET studies in PC3 and AR42J tumor-bearing mice demonstrated that carrier-added **26** showed a 2−3-fold higher tumor uptake at 45–60 min p.i. when compared to background values [56].

Recently, PET imaging of fibrotic diseases, including various types of cancers, by addressing fibrogen activation protein (FAP) by the use of ^68^Ga-labeled FAP inhibitors (FAPI) has gained enormous interest [57,58]. To provide an ^18^F-labeled FAPI for translation into the clinic, the ^18^F-fluoroglycosylation approach by using **14** for click labeling of a FAPI alkyne was reported by Toms et al. [59] The glycoconjugate [^18^F]FGlc-FAPI (**27**, Figure 2) was successfully achieved by the two-step ^18^F-fluoroglycosylation according to Scheme 2, applying optimized reaction conditions for the click labeling step by quenching the deacetylation with phosphate buffer followed by addition of the reactants for ^18^F-fluoroglycosylation at 60 °C for 15 min (Cu(OAc)_2_, THPTA, sodium ascorbate and 400 nmol of FAPI alkyne precursor). For the purpose of preclinical evaluation of **27**, the radiosynthesis was started with 0.5–1 GBq, providing the formulated tracer with a radioactivity yield of 15%, a radiochemical purity of more than 99%, and a molar activity of 30–200 GBq/mmol. The in vitro and in vivo studies of **27** in tumor-bearing mice demonstrated, in direct comparison with [^68^Ga]Ga-FAPI-04, a significantly higher blood protein binding of **27** in vitro, comparable tumor uptake with a high tumor retention and a 2-fold higher blood concentration of **27** in vivo over the 60 min period of the PET scan. Interestingly and in accordance to the higher concentration in blood, **27** showed 2-fold higher specific uptake into murine bone structures and joints compared to [^68^Ga]Ga-FAPI-04. This interesting property could make [^18^F]FGlc-FAPI a candidate ^18^F-labeled FAPI tracer for the imaging of bone tissue remodeling in diseases such as rheumatoid arthritis in humans by PET [59]. Currently, a GMP-compliant automated radiosynthesis of ^18^F-fluoroglycosylated FAPI **27** has been successfully implemented to facilitate first-in-humans PET studies.

## 4. ^18^F-Fluoroglyosylation for the Synthesis of Triazolylalkyl-Linked ^18^F-Glycoconjugates by CuAAC

**Figure 3:**
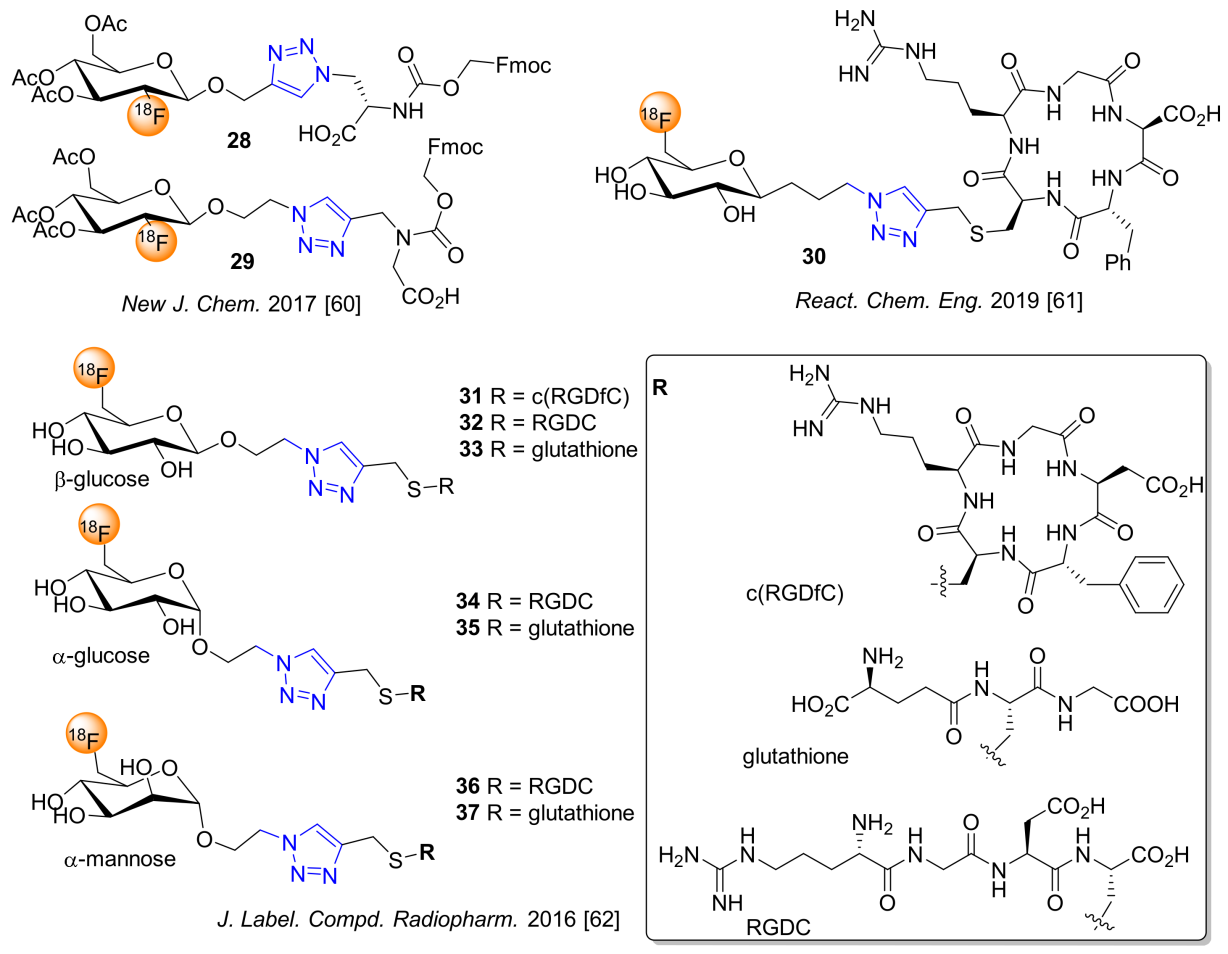
Overview of triazolylalkyl-linked ^18^F-glycoconjugates achieved by click chemistry-based ^18^F-fluoroglycosylation.

The introduction of an alkyl spacer between ^18^F-labeled glycosides and various alkyne-bearing bioactive compounds was achieved by CuAAC, resulting in oxyethyl, oxymethyl, or alkyl linked ^18^F-glycoconjugates (Figure 3). Egland et al. applied the ^18^F-fluoroglycosylation strategy using *O*-alkylated β-mannopyranosides functionalized with a terminal azide or alkyne group to conjugate with _L_-alanine or glycine analogs to give **28** and **29** [60]. The nucleophilic ^18^F-substitution of the β-mannopyranoside precursors was performed with 77–88 % RCY and the ^18^F-labeled glycosides as prosthetic groups were subjected to CuAAC reactions with functional Fmoc-3-azido-l-alanine and Fmoc-*N*-(propargyl)-glycine, which provided the corresponding ^18^F-fluoroglycosylated amino acid conjugates **28** and **29** in high radiochemical yields. The newly synthesized ^18^F-fluoroglycosylated amino acids were used as metabolic radiotracers in PET imaging studies [60].

Collet et al. described the fully-automated radiosynthesis of 6-[^18^F]fluoro-*C*-glyco-c(RDGfC) (**30**) in sequential three steps in a one-pot synthesis, affording the ^18^F-glyco-RGD peptide in high radiochemical purity and a decay-corrected RAY of 3.6% within less than 2.5 h using a fully automated synthesis module. The glycoconjugate **30** showed high stability and hydrophilicity, representing an alternative RGD radiopeptide for imaging integrin expression by PET [61]. Collet et al. further developed a series of 6-[^18^F]fluoro-carbohydrate-based prosthetic groups and their conjugation to glutathione or RGD peptides via click chemistry. In this study, the authors applied ^18^F-fluoroglycosylation by CuAAC reaction with various glycosides, such as β-glucosyl, α-glucosyl and α-mannosyl derivatives bearing the anomeric O-ethyl spacer with terminal azide moiety and a thiol-propargyl moiety attached to RGD peptides or glutathione, affording the ^18^F-glycoconjugates **31–37** (Figure 3) in RCY of up to 76% [62]. A high uptake of 6-[^18^F]fluoro-*O*-glyco-c(RDGfC) (**31**) was shown by PET imaging in rats, revealing the potential of this tracer to monitor integrin expression as part of inflammatory processes and/or angiogenesis.

## 5. Examples of Non-Radioactive Fluoroglycosylation by Click Chemistry and Effects on Inhibitory Potency or Receptor Affinity

**Figure 4:**
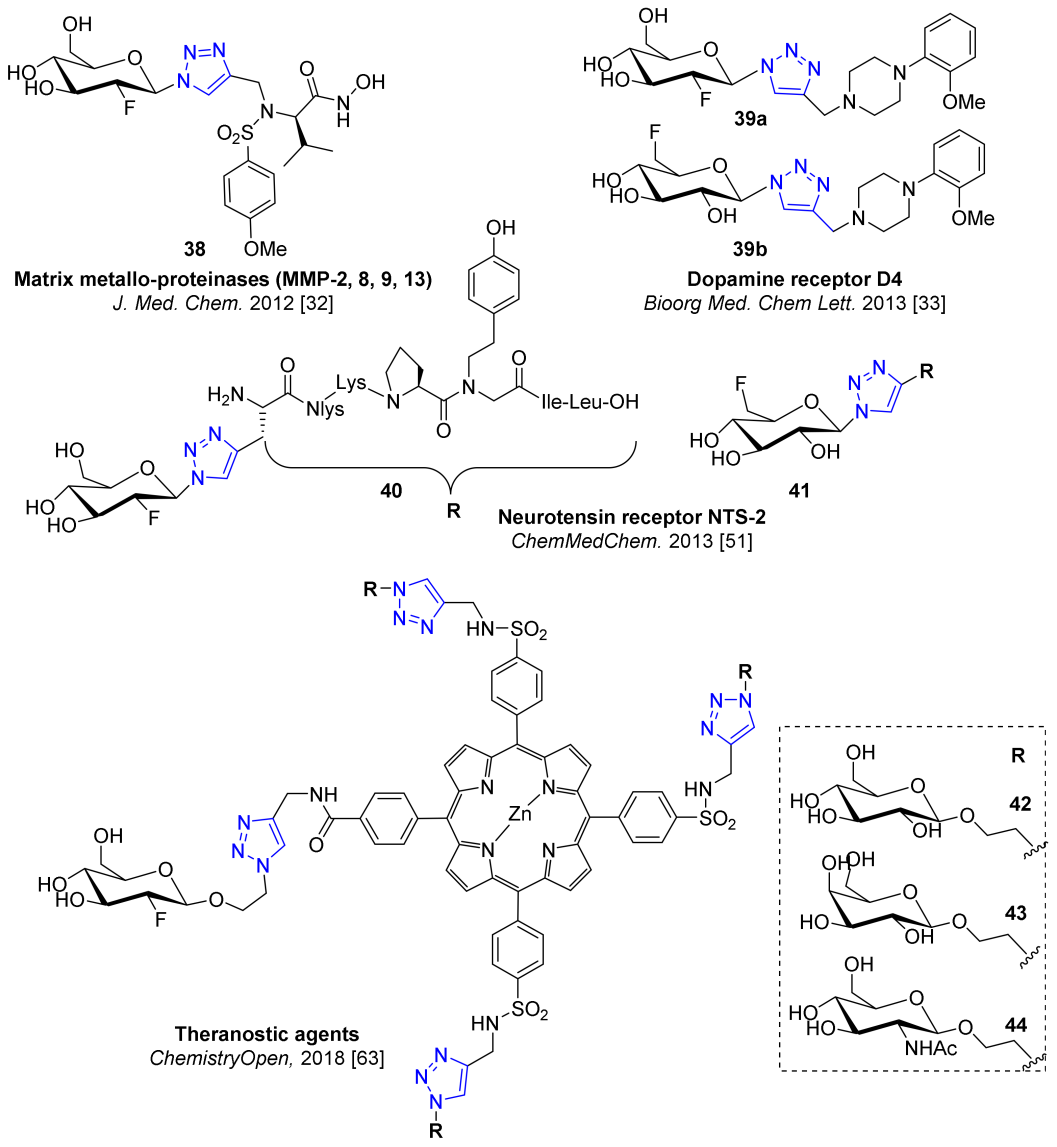
Examples of fluoroglycosylated compounds that have been studied for their biological activity prior to ^18^F-fluoroglycosylation.

Some interesting studies on the effect of fluoroglycosylation on inhibitory potency or receptor affinity are of precedence in the literature (Figure 4). Without any doubt, it is of special importance to study such effects to further improve our knowledge of the effectiveness of ^18^F-fluoroglycosylated tracers for PET. For example, a series of triazolyl-linked inhibitors for the matrix metalloproteinases (MMPs) MMP-2, MMP-8 MMP-9 and MMP-13 as attractive targets for PET were developed by Hugenberg et al. [32]. The fluoroglycosylated compound **38**, which was synthesized by a click chemistry-based method, displayed a logD_7.4_ of 0.58 and subnanomolar inhibition constant of 0.2–0.6 nM. However, the more lipophilic fluoroethyl-1,2,3-triazole analog (clogD_7.4_ = 1.53) revealed outstanding inhibition potencies of 0.006–0.13 nM, therefore rendering the ^18^F-glycoconjugate **38** to be a less suitable PET tracer candidate.

Furthermore, Banerjee et al. reported an example of fluoroglycosylation in their search for subtype selective dopamine D4 receptor radioligands [33], introducing the deoxyfluoroglucosyl compounds **39a** and **39b** (Figure 4). However, the affinities for the D4 receptor with 500 nM and 340 nM, respectively, were 100 to 66 times lower when compared to the fluoropropoxyphenyl compound (5.1 nM), rendering ^18^F-fluoroglycosylation not suitable for this type of ligands.

In addition, the aforementioned study of Held et al. in search of NTS2 selective PET ligands clearly revealed the difference between the introduction of the 2-deoxy-2-fluoroglycosyl and the 6-deoxy-6-fluoroglycosyl moiety to the NTS2-selective Pra-*N*lys-Lys-Pro-*N*-homo-Tyr-Ile-Leu-OH peptide analog, when both **40** and **41** showed a dramatic loss of NTS2 affinity compared to the non-glycosylated compound (110–290 nM vs. 4 nM), while interestingly, **41** demonstrated superior subtype selectivity for NTS2 (350-fold) compared to **40** (11-fold) [51].

Arja et al. reported a fluoroglucosylated porphyrin derivative for the application in photodynamic therapy [63]. The synthesized 2-deoxy-2-fluoro-β-glucosylated porphyrins **42–44** showed fluorescent properties for optical imaging, generated singlet oxygen in vitro and were showed preferred uptake in melanoma cells. These glycoconjugated porphyrins could be promising radiotraces for combined photodynamic therapy and PET imaging studies, when radiolabeled by chemoselective ^18^F-fluoroglycosylation using [^18^F]FDG as prosthetic group.

## 6. [^18^F]FDG for Chemoselective ^18^F-Fluoroglycosylation by Oxime Linkage

**Scheme 3:**
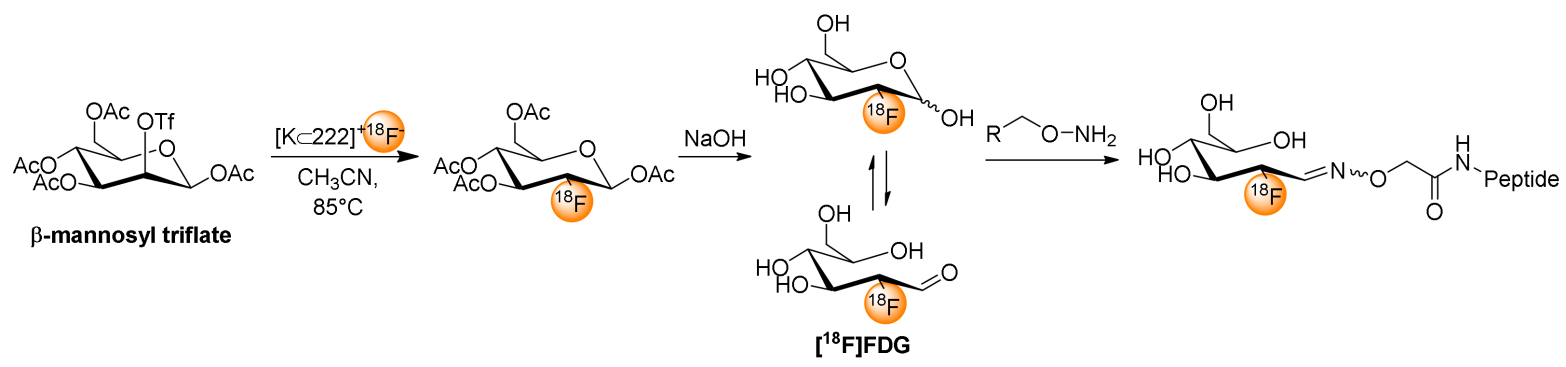
^18^F-fluoroglycosylation via oxime formation using [^18^F]FDG.

The application of aldehyde click chemistry for oxime bond formation in radiopharmaceutical chemistry could be considered as a straightforward alternative method for CuAAC, owing to the favorable properties of oxime bond formation, such as chemoselectivity, high efficiency, and high biocompatibility due to the formation in aqueous solvents [64,65]. Scheme 3 shows the chemoselective oxime formation by click reaction between an aminooxy precursor and the aldehyde functionality of [^18^F]FDG in aqueous solution. [^18^F]FDG could in principle be readily applied to ^18^F-fluoroglycosylation through oxime formation, since in aqueous solution [^18^F]FDG undergoes mutarotation, that is isomerization between the α- and β-anomer via the intermediate acyclic aldehyde, which is favored at high temperatures (80–120 °C) and acidic pH (1.5–2.5).

**Figure 5:**
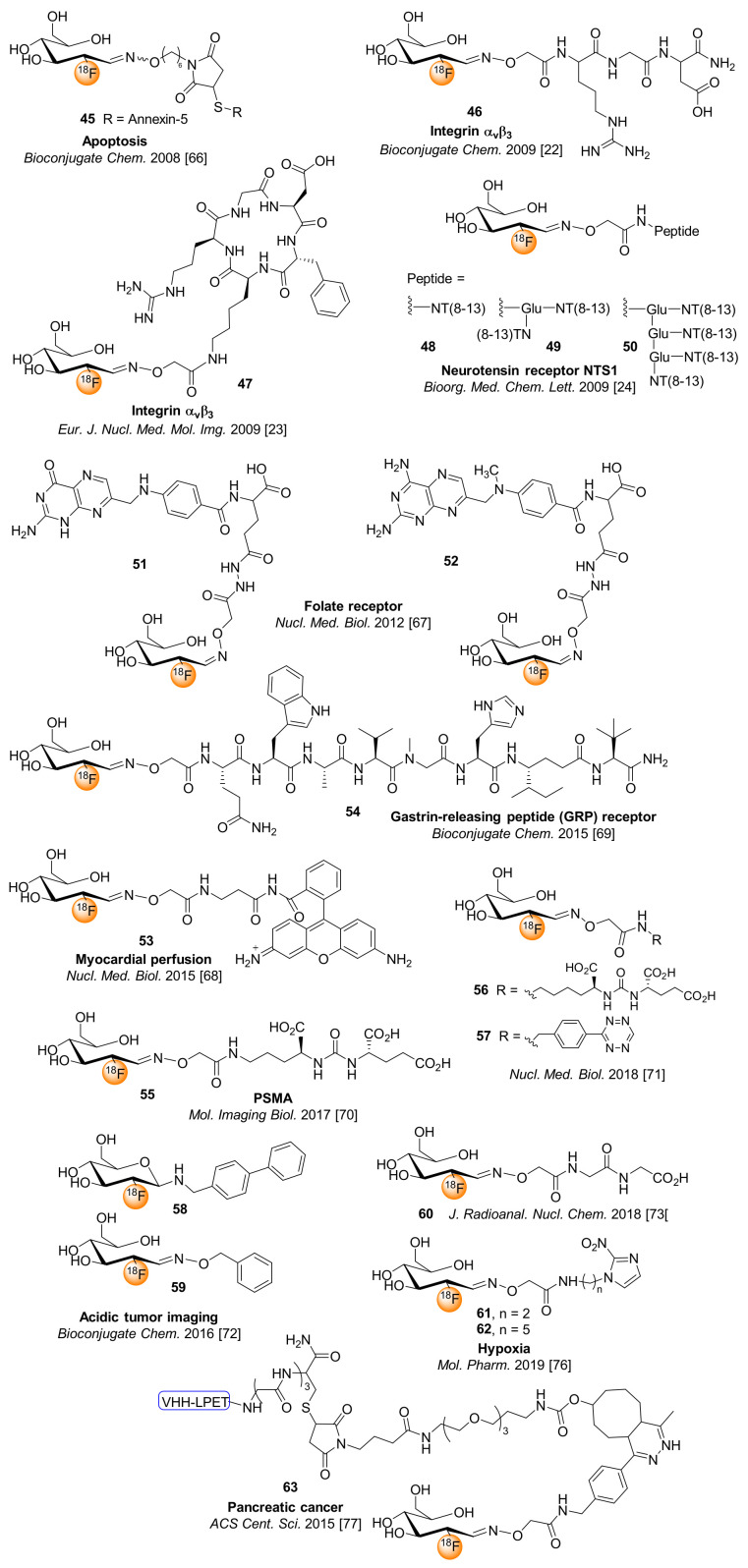
^18^F-glycoconjugates synthesized by direct ^18^F-fluoroglycosylation through oxime formation with [^18^F]FDG.

Alongside the indirect use of [^18^F]FDG for the ^18^F-fluoroglycosylation of thiol-containing peptides through the preparation of a [^18^F]FDG-maleimidehexyloxime prosthetic group ([^18^F]FDG-MHO) [66], the direct ^18^F-fluoroglycosylation of aminooxy-functionalized peptides was first published in 2009 using [^18^F]FDG as prosthetic group [23,24]. These approaches, including the [^18^F]FDG oxime-conjugation with the 36 kDa thiol-group-containing protein annexin-V (**45**) [66] and various aminooxy-functionalized peptides (**46–50** [23,24,25], Figure 5) are discussed in detail in a previous review [21]. Notably, the clinically available [^18^F]FDG solution could not easily been applied for direct ^18^F-fluoroglycosylation of peptide precursors, since the concentration of approximately 0.2 g/mL glucose clearly hampers the ^18^F-fluoroglycosylation reaction, making HPLC purification of [^18^F]FDG prior to use indispensable. Another disadvantage of the ^18^F-fluoroglycosylation by the use of [^18^F]FDG is that high amounts of aminooxy-functionalized peptides (7.5–50 mM) are needed and these precursors often lack stability on storage. However, the direct ^18^F-fluoroglycosylation by oxime formation with [^18^F]FDG is a straightforward approach allowing RCY of up to 80–93% for peptide labeling, providing interesting ^18^F-glycopeptides for PET imaging studies (Figure 5).

Alongside peptides, [^18^F]FDG as a prosthetic group has been applied to the ^18^F-fluoroglycosylation of folate and methotrexate to give conjugates **51** and **52** [67]. The aminooxy-functionalized precursors (9 mM) were conjugated with [^18^F]FDG in DMSO/1% acetic acid/EtOH (1:1, pH∼4.5) at 60 °C for 10–15 min, achieving glycoconjugates **51** and **52** in overall RCY of at least 80%, within a total synthesis time of 20 min and in molar activities of >9 GBq/µmol. The ^18^F-glycoconjugates **51** and **52** displayed favorable binding affinities to folate receptor-positive KB cells when compared to aromatic conjugates and in vivo studies in KB tumor-bearing nude mice showed low uptake in intestine, liver and kidney, rapid clearance from the blood, and high specific uptake of **51** in the tumor, resulting in tumor-to-blood ratio of 11 [67].

More recently, [^18^F]FDG was conjugated to rhodamine by oxime coupling [68]. The radiosynthesis of [^18^F]FDG-rhodamine conjugate **53** was achieved in a simple and convenient way by a one-step process, affording high RCY and 98% radiochemical purity of the formulated tracer after 20 min total synthesis time. Biodistribution studies of **53** in rats revealed an uptake of 11%ID/g in the heart at 60 min p.i., rendering **53** suitable as an imaging agent for the PET evaluation of myocardial perfusion after translation into the clinic.

Richter et al. developed the [^18^F]FDG-conjugated bombesin analog QWAV-Sar-H-FA01010-Tle-NH_2_ ([^18^F]FDG-AOAc-BBN2, **54**) for PET imaging of gastrin-releasing peptide (GRP) receptor-expressing prostate tumors by PET [69]. The bombesin-[^18^F]FDG conjugate **54** provided a favorable pharmacokinetic profile compared to BBN2 conjugated to other ^18^F-labeled prosthetic groups. The ^18^F-glycopeptide **54** revealed high tumor accumulation, fast renal excretion due to low lipophilicity, and high metabolic stability in mouse xenografts using small-animal PET, such that **54** was considered as favorable candidate for imaging GRP-positive prostate cancer by PET.

The Wuest group has described the synthesis and evaluation of PSMA inhibitors conjugated to various ^18^F-labeled prosthetic groups [70]. The ^18^F-fluoroglycosylation of a suitable PSMA derivative with lysins-urea-glutamate scaffold was achieved with [^18^F]FDG via oxime bond formation. The resulting ^18^F-glycoconjugate **55** was isolated by HPLC purification in a decay-corrected RCY of 69% and molar activity of 40 GBq/µmol. Glycoconjugate **55** showed an IC_50_ value of 62 nM for PSMA inhibitory potency, which was a factor of 10 worse than the corresponding fluorophenyl analog. In vivo tumor uptake of the glycoconjugate **55** was similarly inferior by a factor of 10 compared with the fluorophenyl analog, as demonstrated by dynamic PET studies in LNCaP tumor-bearing mice [70].

To avoid the HPLC purifications after ^18^F-fluoroglycosylation via oxime formation, Keinänen et al. developed solid-phase extraction and resin purification protocols for the synthesis of glycoconjugates **56** and **57** (Figure 5) [71]. The purification of the final ^18^F-glycoconjugates was achieved by removal of unreacted carbohydrate via derivatization with 4,4′-dimethoxytrityl chloride (DMT-Cl) and the removal of excess aminooxy precursors after ^18^F-fluoroglycoconjugation was achieved by the use of an aldehyde resin (AminoLink).

Flavell et al. developed an [^18^F]FDG amine prodrug targeting tissue regions with low interstitial pH [72]. The [^18^F]FDG-derived glycosylamine **58** was synthesized in one step from [^18^F]FDG by treatment with 4-phenylbenzylamine in acidic acid at 80 °C. The resulting ^18^F-glycosylamine **58** was isolated in 20% RAY and showed greater uptake in tumor tissue relative to benign tissue, revealing favorable and pH-dependent properties for tumor uptake when compared to the oxime-linked analog **59** (Figure 5). Therefore, the ^18^F-glycosylamine **58** could be a promising acid-responsive PET tracer for tumor imaging.

The very simple procedure for using clinically readily available FDG for ligation with peptides was demonstrated using the small peptide glycylglycine as an example [73]. Starting from the commercially available [^18^F]FDG solution and after cleavage of the BOC protecting group from the aminooxy-derivatized peptide, [^18^F]FDG-GlyGly (**60**) could be obtained in a RCY of 98% at 100 °C after 30 min.

The PET imaging of tumor hypoxia using 2-nitroimidazole tracers is well established in nuclear medicine practice [74]. By using an approach similar to that described by Patt et al. [75], Yang et al. synthesized the [^18^F]FDG-conjugated 2-nitroimidazole **61** and **62** via oxime bond formation, introducing ethyl and pentylaminecarbamate alkyl chains as spacers with different lengths between the glycosyl moiety and the nitroimidazole [76]. The biodistribution studies revealed that compound **61** (ethyl spacer) showed better in vivo properties than compound **62** (pentyl spacer), probably due to its lower lipophilicity.

Rashidian et al. developed the ^18^F-labeled single domain antibody fragment for PET imaging of pancreatic tumors in mice [77]. [^18^F]FDG was coupled to a tetrazine scaffold by oxime ligation and subsequently conjugated to the trans-cyclooctene (TCO)-functionalized peptide VHH-LPET via TCO-tetrazine sortase-mediated reaction, affording the ^18^F-glycosylated antibody fragment **63** (Figure 5) after 20 min of constant agitation at 25 °C. Conjugate **63** showed promising characteristics for the detection of the growth and regression of small pancreatic tumors by immune-PET imaging.

## 7. 5-[^18^F]fluoro-5-deoxyribose ([^18^F]FDR) for Chemoselective ^18^F-fluoroglycosylation by Oxime Linkage

**Scheme 4:**
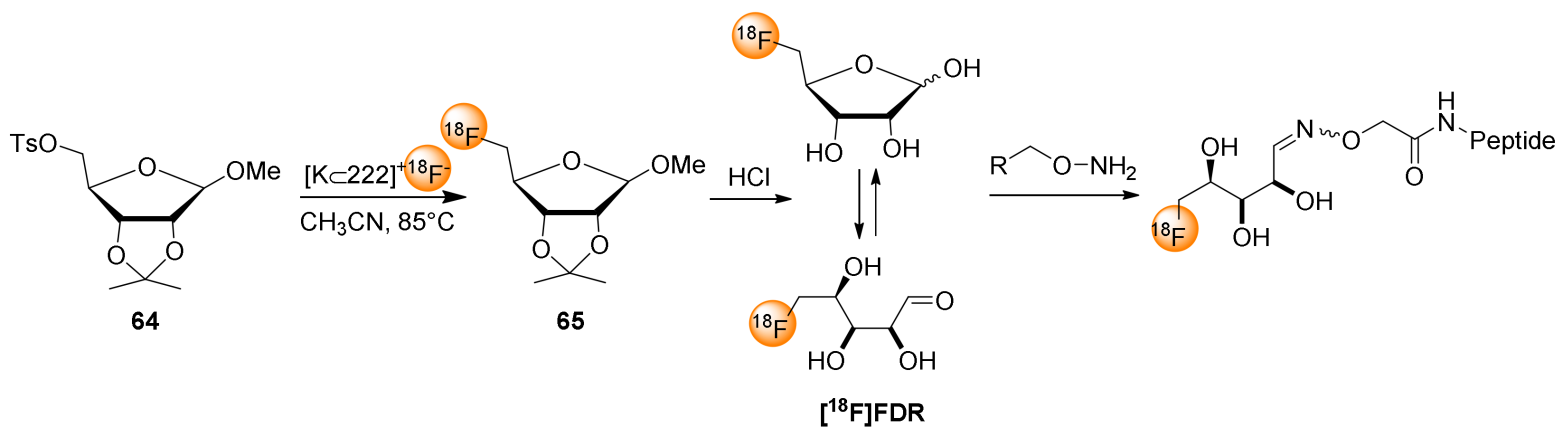
^18^F-fluoroglycosylation via oxime formation using [^18^F]FDR.

The use of [^18^F]FDG for direct ^18^F-fluoroglycosylation via oxime formation requires relatively harsh reaction conditions, namely high temperature and acidic pH, which is unfavorable for sensitive biomolecules. To overcome this limitation, 5-[^18^F]fluoro-5-deoxyribose ([^18^F]FDR) can be considered as an alternative prosthetic group for ^18^F-fluoroglycosylation, because the location of the fluorine at C-5 of the 5-deoxyribose ring facilitates the formation of the acyclic form of [^18^F]FDR making oxime bond formation possible at ambient temperature and pH of 4–5 [65,78]. Scheme 4 shows the aminopolyether-supported radiosynthesis of [^18^F]FDR starting from methyl 2,3-*O*-isopropylidene-5-*O*-(*p*-toluenesulfonyl)-β-d-ribofuranoside (**64**) and the subsequent oxime bond formation with aminooxy-functionalized peptides.

Noteworthily, HPLC separation of the intermediate methyl 2,3-*O*-isopropylidene-5-deoxy-5-[^18^F]fluororibofuranoside (**65**) from excess precursor **64** turned out to be essential for the efficient use of [^18^F]FDR in subsequent ^18^F-fluoroglycosylation reactions. After the acidic hydrolysis of **65** and solid phase extraction, [^18^F]FDR was obtained in average at 35% RCY with a total synthesis time of 85 min [27,78].

**Figure 6:**
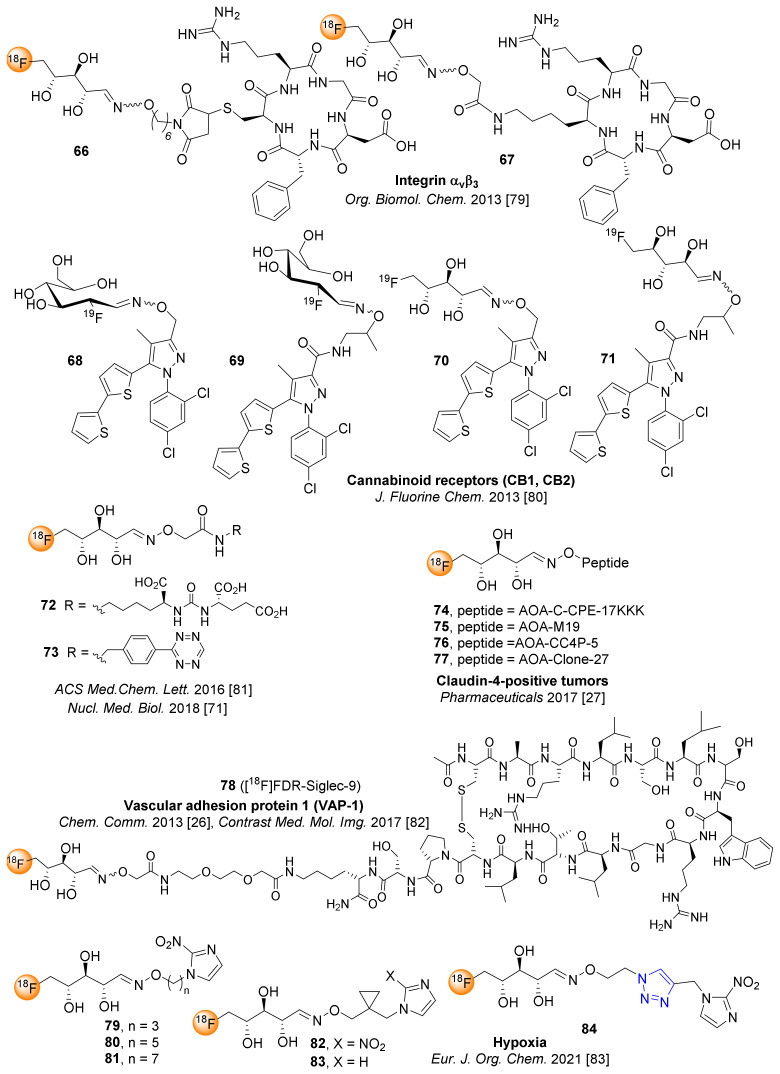
^18^F-glycoconjugates synthesized by oxime formation with [^18^F]FDR.

[^18^F]FDR was conjugated to the aminooxy-functionalized RGD peptides c(RGDfK) and c(RGDfC) at room temperature in sodium acetate buffer at pH 4.6, affording 65–92% RCY in 15 min [79]. The resulting ^18^F-glycopeptides **66** and **67** (Figure 6) were isolated by radio-HPLC and showed specific binding to α_v_β_3_-expressing PC3 cells, demonstrating that [^18^F]FDR is an effective prosthetic group for ^18^F-fluoroglycosylation of bioactive RGD peptides [79].

In a comparative study of non-radioactive FDR and FDG for oxime formation with hydroxylamine-functionalized rimonabant-type pyrazoles, glycoconjugates **68–71** were synthesized as candidate PET ligands for cannabinoid receptors 1 (CB1) and 2 (CB2) [80]. As expected, FDR conjugation proved to be superior to FDG analogues, as the conjugation proceeded at room temperature in 20 min, whereas FDG conjugation required 100 °C (30 min). However, **68–71** showed only weak affinities to CB1 (540–720 nM) and CB2 (310–1400 nM), such that subsequent studies on ^18^F-glycosylation were not reasonable.

Alongside the [^18^F]FDG conjugates **56** and **57** (see Figure 5), Keinänen et al. reported the [^18^F]FDR-conjugated PSMA inhibitor **72** (Figure 6) and tetrazine analog **73** as a prosthetic group for inverse electron-demand Diels–Alder cycloaddition (IEDDA) reactions with trans-cyclooctene derivatives, being compatible for pretargeted in vivo PET imaging studies [81]. The ^18^F-glycosylated tetrazine **73** showed low lipophilicity and excellent stability in phosphate-buffered saline and in mouse plasma. The biodistribution study of **73** in mice demonstrated promising pharmacokinetics that could be suitable for in vivo bioorthogonal IEDDA reactions in future pre-targeted PET imaging studies. The reported solid-phase purification method applied for both [^18^F]FDG- and [^18^F]FDR-conjugated products, providing **56**, **57** and **72**, **73** in high radiochemical purity and molar activity (Figure 5 and 6) [71,81].

The Neumaier group synthesized and studied various ^18^F-labeled peptides for imaging of claudin-4 as candidate tracers for PET imaging of pancreatic tumors [28]. The various [^18^F]FDR-conjugated peptides **74–77** were synthesized via oxime ligation of claudin-derived peptides, applying [^18^F]FDR obtained by ^18^F-labeling of the naphthalene onium salt of 5-deoxyribose (Table 1). The ^18^F-glycosylated peptide **77** (Figure 6) was afforded in high radiochemical purity (>98%) and 15% RCY after a total synthesis time of 98 min, successfully introducing a “minimalist” protocol for ^18^F-synthesis by taking advantage of the onium salt precursor [28].

Li et al. demonstrated the ^18^F-fluoroglycosylation by the use of [^18^F]FDR using sialic acid-binding Ig-like lectin 9 (siglec-9) [26], a protein ligand for vascular adhesion protein 1 (VAP-1) which is upregulated in inflammation. Since siglec-9 is a rare temperature sensitive peptide, the authors optimized the ^18^F-fluoroglycosylation with [^18^F]FDR by the use of an anilinium buffer (pH 4.6) instead of sodium acetate, to allow oxime bond formation at a minimized peptide concentration of 0.3 mM, affording the desired ^18^F-glycopeptide with 50–60% RCY after ligation for 10 min at room temperature. [^18^F]FDR-Siglec-9 (**78**) was formulated within 120 min after the final HPLC purification with a RAY of 27% and a molar activity of 36–43 GBq/µmol. In vivo experiments clearly demonstrated that **78** could be successfully applied for the detection of inflammatory foci in rats [27]. Moreover, the glycoconjugate **78** was compared with ^68^Ga-DOTA-Siglec-9, revealing very similar tracer properties for the detection of inflammatory lesions in vivo [82]; however, since the radiosynthesis of **78** turned out to be more laborious and time-consuming, the ^68^Ga-DOTA-conjugated Siglec-9 analog was suggested as more advantageous for future clinical studies.

More recently, Musolino et al. reported the synthesis of radiotracers **79–84** for detection of hypoxia cells using PET [83]. The hypoxia-reactive 2-nitroimidazoles, bearing different alkyl chains or triazole moieties as spacers, were conjugated to [^18^F]FDR via oxime linkage. Interestingly, the introduction of the cyclopropyl ring in the spacer (**82**, Figure 6) showed superior uptake kinetics and selectivity for hypoxia cells.

## 8. Miscellaneous ^18^F-fluoroglycosylation Reactions

There are alternative ^18^F-glycosylation strategies present in the literature, consisting of the early works that apply tetraacetylated FDG, the intermediate of the FDG synthesis, as ^18^F-glycosyl donor in the presence of Lewis acids or Koenigs–Knorr conditions [75,84,85], alternative approaches toward thiol-selective ^18^F-fluoroglycosylation [66,83,86], the use of thiol-reactive FDG derivative for ^18^F-labeling of magnetic nanoparticles for combined PET/MR studies [87,88], and rarely studied enzymatic ^18^F-fluoroglycosylation reactions [89,90] or, more recently, the use of acidic reaction conditions for the direct ligation of [^18^F]FDG to 4-amino-phenylalanine in 79% RCY [91], providing a novel ^18^F-glycosylated amino acid PET tracer that could be valuable for the differentiation of tumor tissue from inflammatory lesions in future clinical studies.

## 9. Conclusions and Future Prospects

The number of literature examples for the application of ^18^F-fluoroglycosylation as a strategy for the successful development of PET tracers has increased further since 2014. Above all, the biocompatible methods of the mild chemoselective click chemistry conjugations are preferred in most cases. Almost every suitable ^18^F-glycosylated tracer described in the literature has high stability in vivo, and very good clearance properties in vivo, whereby the clearance through kidneys can be significantly influenced by the position of ^18^F-substitution in the carbohydrate ring.

In particular, lipophilic receptor ligands gain hydrophilicity and lose blood protein binding by ^18^F-fluoroglycosylation. Therefore, ^18^F-labeling and concomitant glycosylation for lipophilic ligands is a very suitable method for the development of PET ligands, provided that receptor binding is not affected by glycosylation. Furthermore, ^18^F-fluoroglycosylation is particularly well suited for the derivatization of peptides, since suitable alkyne-modified amino acids are commercially available for the targeted introduction to solid-phase peptide synthesis and the binding properties of resulting glycopeptides are thus very little or not affected at all.

The GMP-compliant automated two-step ^18^F-fluoroglycosylation has been established for promising ^18^F-fluoroglycosylated tracers and is being further improved to enable the translation of ^18^F-glycoconjugates into the clinic. However, the difficulty to be overcome with respect to the automation of ^18^F-fluoroglycosylation is mainly the two-step and thus laborious synthesis. In the future, it will be necessary to shorten and simplify the synthesis of ^18^F-glycoconjugates, for example by using solid-phase bound glycosyl precursors. In particular, for ^18^F-glycopeptide tracers or [^18^F]FGlc-FAPI (**27**), the improved availability could significantly promote and facilitate the use of these glyco-tracers in first-in-humans studies in the future.

## Figures and Tables

**Table 1:** Overview of a selection of ^18^F-labeled prosthetic groups for ^18^F-fluoroglycosylation reactions.

Labeling Precursor	Prosthetic Group	Reaction Conditions	Ref.
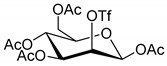	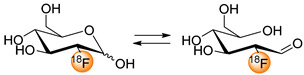	1. K_222_, K_2_CO_3_ 2. NaOH	[23,24,25]
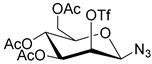	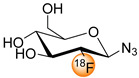	1. K_222_, K_2_CO_3_ 2. NaOH	[19]
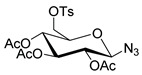	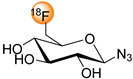	1. K_222_, K_2_CO_3_, KH_2_PO_4_ 2. NaOH	[26]
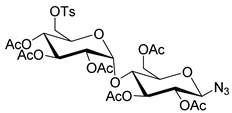	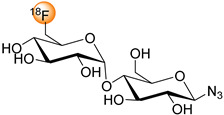	1. K_222_, K_2_CO_3_, KH_2_PO_4_ 2. NaOH	[26]
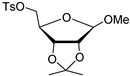		1. K_222_, K_2_CO_3_, 2. DMT-Cl, pyridine 3. HCl	[27]
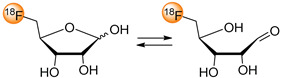	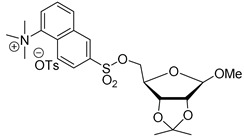	1. MeCN, 120 °C 2. 1M HCl, 110 °C	[28]

## Data Availability

Data sharing not applicable.
